# Serum levels of biomarkers related to severity staging of Raynaud’s phenomenon, neurosensory manifestations, and vibration exposure in patients with hand-arm vibration injury

**DOI:** 10.1038/s41598-024-68846-1

**Published:** 2024-08-05

**Authors:** Eva Tekavec, Tohr Nilsson, Lars B. Dahlin, Elizabeth Huynh, Catarina Nordander, Jakob Riddar, Monica Kåredal

**Affiliations:** 1 Division of Occupational and Environmental Medicine, Department of Laboratory Medicine, Lund University, 221 00 Lund, Sweden; 2 Division of Sustainable Health and Medicine, Department of Public Health and Clinical Medicine, Umeå University, 901 87 Umeå, Sweden; 3 Department of Translational Medicine – Hand Surgery, Lund University, 221 00 Lund, Sweden; 4 Department of Occupational and Environmental Medicine, Region Skåne, 223 63 Lund, Sweden

**Keywords:** Hand-arm vibration syndrome (HAVS), Vibration exposure, Occupational, Serum biomarkers, Grading of injury, Endothelial dysfunction, Neuroprotection, C-fibers, Aδ fibers, Aβ fibers, Cold intolerance, Biomarkers, Health occupations, Medical research, Pathogenesis, Risk factors, Signs and symptoms

## Abstract

Our aim was to explore possible relationships between serum levels of biomarkers in patients with hand-arm vibration injury in relation to the severity of the vascular, i.e., Raynaud’s phenomenon (RP), and neurosensory manifestations, the current exposure level, and the duration of exposure. This study was of case series design and involved 92 patients diagnosed with hand-arm vibration injury. Jonckheere’s trend test was used to assess any association between serum levels of biomarkers and RP as well as neurosensory manifestations, graded by the International Consensus Criteria. Generalized linear models with adjustment for possible confounders were also used for associations between serum levels of biomarkers and; (1) severity of RP recorded as the extent of finger blanching calculated with Griffin score, (2) vibration perception thresholds, (3) magnitude of current exposure as [A(8); (m/s^2^)] value, and (4) the duration of exposure in years. Serum levels of thrombomodulin, von Willebrand factor, calcitonin gene related peptide (CGRP), heat shock protein 27, and caspase-3 were positively associated with severity of RP. Serum levels of CGRP were positively associated with the neurosensory component. No associations with exposure were shown for these biomarkers**.** For Intercellular adhesion molecule 1 and monocyte chemoattractant protein 1, no associations were found with neither severity nor exposure. Levels of serum biomarkers associated with endothelial injury or dysfunction, inflammation, vasodilation, neuroprotection, and apoptosis were positively associated with the severity of hand-arm vibration injury.

## Introduction

Hand arm-vibration injury affects many workers globally. Manifest, the condition has a poor prognosis, with little expected improvement even if the exposure ceases^1^. The clinical investigation of vibration-exposed patients is mainly based on medical history, semi-objective findings, exposure assessment, and ruling out other medical conditions with the same clinical presentation^2^. Typical symptoms are episodic finger blanching, Raynaud’s phenomenon (RP), when the worker is exposed to cold or stress, i.e., and symptoms from affected sensory units with small- and large nerve fibers in the fingers and hand-wrist area. Nerve entrapments, e.g., median or ulnar nerve compression, are also part of the diagnosis and sometimes challenge the clinical assessment^1^. In a quest to find objective diagnostic tools in the clinical investigation of these patients, we recently conducted a study on serum biomarkers and the correlation to hand-arm vibration injury^3^.

Studies suggest that pathophysiological mechanisms involve both localized, structural injury to blood vessels and peripheral nerves^4–9^, systemic imbalance of the autonomic nervous system^10^ with effect on the heart rate variability^11,12^, and inflammatory processes via oxidative stress^6,8,13–16^. For example, elevated plasma levels of intercellular adhesion molecule 1 (ICAM-1), a transmembrane protein that is being upregulates under conditions of stress or injury, associated with connective tissue diseases^17^ and correlated to disease activity in patients with RP due to scleroderma^18^ have been shown in an experimental study in vibration exposed individuals^19^ and in vibration injured patients compared to controls^20^.

In the previous study^3^, we found elevated serum levels of biomarkers that are associated with *inflammation, endothelial injury or dysfunction* i.e., intercellular adhesion molecule 1 (ICAM-1), monocyte chemoattractant protein 1 (MCP-1), thrombomodulin (TM), von Willebrand factor (vWf), *vasoregulatory mechanisms i.e.,* calcitonin gene related peptide (CGRP), *neuroprotection i.e.,* heat shock protein 27 (HSP27) and *apoptotic mechanisms i.e.,* caspase-3, in patients with hand-arm vibration injury compared to controls. Furthermore, serum levels of TM, vWf and CGRP were elevated in patients with RP compared to those without RP.

Whether serum levels of different biomarkers vary with severity of the vascular or neurosensory components of injury is, however, not clear. Elevated plasma levels of TM and ICAM-1 with higher grades on the Stockholm workshop scale (SWS) and with vascular and neurosensory components combined have been reported^20^. SWS is the commonly used system for grading severity of hand-arm vibration injury^21^. The International Consensus Criteria (ICC) is an updated grading system for hand-arm vibration injury^22^. For the vascular component, SWS is based on a combination of the number of affected phalanges and the frequency of finger blanching episodes, which in turn may vary due to climate, outdoor jobs etc. ICC is based solely on the number of affected phalanges, giving more weight to proximal than distal phalanges^23^. The neurosensory component (ICC N) has a stricter criterion for grade 2 and 3 than the SWS SN, requiring more semi-objective findings.

Experimental studies have shown histopathological changes of blood vessels and nerve structures and varying levels of several blood biomarkers^9^ in relation to frequency, magnitude, and duration of the vibration exposure, and the use of impact tools^8,24–29^. Whether and how serum levels of specific biomarkers vary with the magnitude and duration of exposure, is, however, not fully clarified.

Our aim was to explore possible relationships between serum levels of biomarkers in patients with hand-arm vibration injury in relation to the severity of Raynaud’s phenomenon and neurosensory manifestations, the current vibration exposure level, and the duration of exposure.

## Results

Descriptive characteristics (e.g., age, gender, current smoking) are summarized in Table 1. The term “concurrent diseases” includes cardiovascular disease (20%), diabetes (7%), thyroid disease (5%), polyneuropathy (4%), rheumatic disease (0%) and treatment for attention deficient disorder or migraine (4%). Definition of “cardiovascular disease” includes reporting of coronary heart disease, medication with antihypertensive drugs: ACE-inhibitor (Enalapril, Triatec) 11%, angiotensin-II antagonist (Candesartan, Losartan) 7%, calcium-antagonists (Amlodipine, Felodipine) 4%, selective beta-receptor antagonist (Metoprolol, Bisoprolol) 3%, or treatment with Acetylsalicylic acid or Clopidogrel 3%, anti-factor Xa (Xarelto) 2%, Warfarin 1%. Individuals with diabetes reported treatment with Insulin in 4% or Metformin in 3%. ADHD medication (Methylphenidate) was reported in 3%, and migraine medicine (Zolmitriptan) in 2%.

**Table 1:** Descriptive characteristics, severity of vascular (Griffin score) and neurosensory manifestations (vibration perception threshold, VPT) in the hand with most pronounced symptoms, and hand-arm vibration exposure, in 92 patients with hand-arm vibration injury.

Age (years)	45 (21–64)
Females	6 (7)
Ongoing cigarette smoking	14 (15)
Previous frostbites	10 (11)a
Concurrent diseases	27 (29)
Cardiovascular disease	18 (20)
Diabetes	7 (8)
Thyroid diseases	5 (5)
Rheumatic disease	0 (0)
Polyneuropathy	4 (4)
ADHD or migraine medication	4 (4)
Griffin score	0 (0–33)b
Vibration perception thresholds (VPT) with sensibility index (SI)	1.0 (0.4–1.6)
Current exposure [A(8); (m/s^2^)]	2.8 (0.0–7.0)
Duration of exposure (years)	20 (2.6–46)

Data are median (range) or n (%).

^a^Missing data in 7 patients.

^b^Missing data in 10 patients.

**Figure 1:**
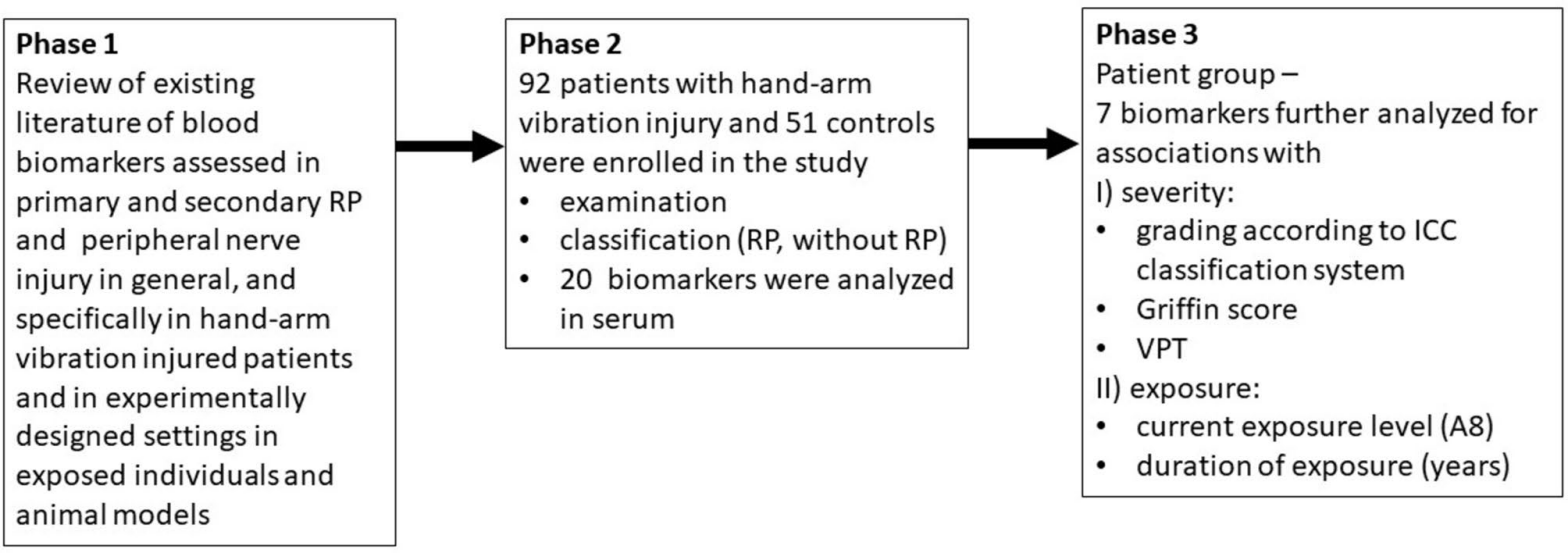
The scheme of the studies.

**Figure 2:**
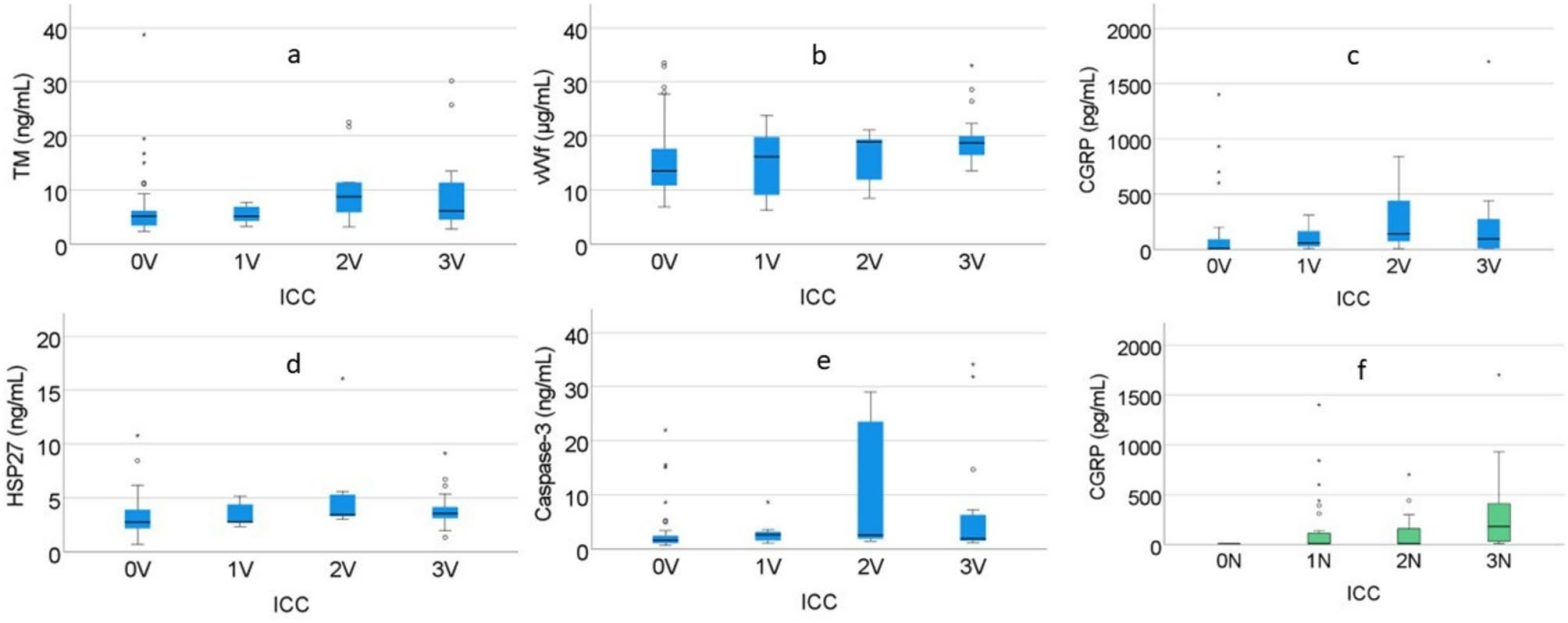
Distribution of serum levels of (**a**) thrombomodulin (TM), (**b**) von Willebrand factor (vWf), (**c**) and (**f**) calcitonin gene-related peptide (CGRP), (**d**) heat shock protein 27 (HSP27), (**e**) caspase-3 in relation to severity of vascular (blue boxes, 82 patients) and sensorineural manifestations (green boxes, 92 patients), as graded by the International Classification Criteria ICC. Medians are indicated with a line. Boxes represent interquartile ranges. Values that are more than 1.5 (circles) or 3 times (stars) higher than the third quartile are indicated as outliers. Error bars indicate min–max values that are not outliers. One patient with S-CGRP 3700 pg/mL; ICC 2 V and 1N, one with S-HSP27 45 ng/mL; ICC 0 V, one with caspase-3 85 ng/mL; ICC 0 V and one with caspase-3 61 ng/mL; ICC 3 V, are not shown as the axes are cut.

Serum levels of TM, vWf, CGRP, HSP27, and caspase-3 were positively associated with severity of RP according to ICC vascular staging^22^ (Table 2, Fig. 2). For several of the biomarkers, as shown in Fig. 1, the positive trends were seen between grade 1 and 2, but not for grade 3. For ICAM-1 and MCP-1 there was no such trend (Table 2). There were linear associations between the log transformed levels of serum levels of TM, vWf and caspase-3 and the severity of RP, (Table 3), which remained after adjustment for possible confounding. For HSP27 the association was not linear.

**Table 2:** Serum levels of biomarkers in 92 patients with hand-arm vibration injury according to the severity of vascular manifestations graded by ICC 0–3 V.

Biomarkers	ICC 0 V n = 47	ICC 1 V n = 7	ICC 2 V n = 9	ICC 3 V n = 19	*P* value^a^
ICAM-1 (ng/mL)	170 (150–200)	180 (170–210)	170 (170–190)	170 (150–180)	0.59
MCP-1 (pg/mL)	39 (30–57)	41 (33–58)	42 (32–76)	36 (26–48)	0.50
TM (ng/mL)	5.2 (3.4–6.2)	5.1 (4.2–7.1)	8.7 (5.8–17)	6.1 (4.5–12)	**0.01**
vWf (µg/mL)	14 (6.9–34)	16 (7.6–23)	19 (11–20)	19 (16–21)	**0.007**
CGRP (pg/mL)	< LOD^b^ (< LOD–96)	58 (< LOD–220)	140 (40–640)	94 (< LOD–300)	**0.001**
HSP27 (ng/ml)	2.8 (2.1–4.0)	2.8 (2.7–4.9)	3.4 (3.2–5.5)	3.6 (3.1–4.4)	**0.007**
Caspase-3 (ng/mL)	1.6 (1.0–2.5)	2.6 (1.1–3.6)	2.6 (1.8–24)	1.9 (1.4–7.2)	**0.007**

^a^Median (interquartile ranges).

^b^10 individuals were excluded since they had not filled in hand diagram.

^c^*P* values in boldface denotes statistically significant associations with Jonckheere-Tepstra trend test.

^d^Limit of detection (LOD) 15 pg/ml.

**Table 3:** Associations (β with 95% confidence intervals) between log transformed values in serum biomarkers and severity of hand-arm vibration injury, estimated by Generalized Linear Models in 92 patients with hand-arm vibration injury. Statistically significant associations in bold face.

Biomarker	Raynaud’s phenomenon (Griffin score) n = 82		Vibration perception threshold (sensibility index) n = 92	
	Crude	Adjusted	Crude	Adjusted
ICAM-1	− 0.00 (− 0.01 to 0.00)		− 0.00 (− 0.13 to 0.14)	
MCP-1	− 0.01 (− 0.02 to 0.00)	− 0.01 (− 0.02 to 0.00)^a^	− 0.21 (− 0.60 to 0.18)	− 0.25 (− 0.64 to 0.14)^a^
TM	**0.01 (0.00–0.03)**		− 0.26 (− 0.76 to 0.23)	
vWf	**0.01 (0.00–0.02)**	**0.01 (0.00–0.02)**^b^	− 0.06 (− 0.37–0.26)	
Without concurrent diseases				− 0.23 (− 0.55 to 0.08)^c^
With concurrent diseases				0.59 (− 0.07 to 1.3)^c^
HSP27	0.01 (− 0.00–0.02)	0.01(− 0.01 to 0.02)^d^	− 0.04 (− 0.52 to 0.43)	− 0.01 (− 0.45 to 0.46^d^
Caspase-3	**0.03 (0.00–0.05)**	**0.03 (0.01–0.05)**^e^	− 0.44 (− 1.3 to 0.47)	
Without concurrent diseases				− **1.1 (**− **2.1 to 0.09)**
With concurrent disease				1.4 (0.28–3.1)

^a^Adjusted for current smoking and frostbites.

^b^Adjusted for age and concurrent diseases.

^c^Adjusted for age.

^d^Adjusted for age, concurrent diseases, and sex.

^e^Adjusted for concurrent diseases.

Serum levels of CGRP were positively associated with the neurosensory component, graded by ICC 0-3N (Table 4, Fig. 2). There were, however, no linear associations between the log transformed levels of any of the biomarker and vibration perception thresholds (Table 3), nor the with the current exposure or duration of exposure (Table 5).

**Table 4:** Serum levels of biomarkers in 92 patients with hand-arm vibration injury according to the severity of neurosensory manifestations graded by ICC 1-3N.

Biomarkers	ICC 1N n = 39	ICC 2N n = 36	ICC 3N n = 15	*P* value^a^
ICAM-1 (ng/mL)	170 (150–190)	170 (160–200)	170 (150–170)	0.42
MCP-1 (pg/mL)	39 (31–54	38 (30–52)	39 (31–64)	0.76
TM (ng/mL)	5.4 (4.2–7.7)	4.5 (3.3–7.0)	6.6 (5.1–12)	0.51
vWF (µg/mL)	15 (12–19)	16 (11–22)	16 (13–19)	0.47
CGRP (pg/mL)	< LOD^b^ (< LOD–120)	< LOD (< LOD–170)	180 (< LOD–420)	**0.04**
HSP27 (ng/ml)	2.9 (2.3–4.3)	3.1 (2.4–3.8)	3.4 (3.1–5.2)	0.21
Caspase-3 (ng/mL)	1.8 (1.2–3.6)	1.7 (1.2–2.8)	2.2 (1.2–15)	0.45

Median (interquartile ranges).

Stage 0N is not included (n = 2).

^a^*P* values in boldface denotes statistically significant associations, with Jonckheere-Tepstra trend test.

^b^Limit of detection (LOD) 15 pg/ml.

**Table 5:** Associations (β with 95% confidence intervals) between log transformed values in serum biomarkers and severity of hand-arm vibration exposure, estimated by Generalized Linear Models in 92 patients with hand-arm vibration injury.

Biomarker (ln)	Current vibration exposure [A(8); (m/s^2^)] n = 92		Duration of exposure (years) n = 92	
	Crude	Adjusted	Crude	Adjusted
ICAM-1 (ng/mL)	− 0.01 (− 0.03 to 0.02)		0.00 (− 0.00 to 0.00)	
MCP-1(pg/mL)	− 0.06 (− 0.12 to 0.01)	− 0.07 (− 0.14 to − 0.01)^a^	0.00 (− 0.01 to 0.01)	0.00 (− 0.01 to 0.01)^a^
TM (ng/mL)	− 0.03 (− 0.12 to 0.05)		0.00 (− 0.01 to 0.01)	
vWf (µg/mL)	− **0.06** (− **0.12** to − **0.01**)	− 0.04 (− 0.09 to 0.01)^b^	**0.01 (0.01 **to** 0.02)**	0.01 (− 0.00 to 0.02)^b^
HSP-27 (ng/ml)	0.02 (− 0.07 to 0.10)	0.04 (− 0.05 to 0.12)^d^	0.01 (− 0.00 to 0.02)	− 0.01 (− 0.02 to 0.01)^d^
Caspase-3 (ng/ml)	0.01 (− 0.14 to 0.17)		− 0.00 (− 0.02 to 0.02)	− 0.00 (− 0.02 to 0.02)^e^
Without concurrent diseases		− 0.24 (− 0.46 to − 0.03)		
With concurrent diseases		0.20 (0.00 to 0.40)		

Statistically significant associations in bold face.

Adjustment for possible confounders with an association to the biomarker. For confounders with interaction, data was dichotomised according to that factor:

^a^Adjusted for current smoking and previous frostbites.

^b^Adjusted for age and concurrent disease.

^c^Adjusted for sex.

^d^Adjusted for age, concurrent diseases, and sex.

^e^Adjusted for concurrent diseases.

## Discussion

Serum levels of TM, vWf, HSP27, CGRP and caspase-3 were positively associated with the severity of Raynaud’s´ phenomenon according to ICC vascular staging. TM, vWf and caspase-3 showed a linear association, while for HSP27, there was also a positive association, though not linear. In addition, increased serum levels of CGRP were associated with increased severity of neurosensory manifestations according to ICC staging. No linear associations were, however, observed between log transformed levels of any serum biomarker and VPT, current exposure level or duration of exposure.

A limitation of the study was that the episode of finger blanching was assessed based only on medical history. Apart from this, RP was graded according to guidelines in the ICC and established on patients’ history of number of phalanges affected on a “typical” episode of finger blanching. This gives an estimate of severity that does not depend on climate or whether the patient works indoors or outdoors, which makes the estimate more reliable. Another possible limitation could be that we did not include patients using serotonin or noradrenalin reuptake inhibitors and statins, but with no other treatment for cardiovascular disease, in our definition of patients with concurrent disease. No power calculation was performed prior to the study since it was not possible to predict an effect size since reference values for the selected biomarkers are vaguely defined. The question is relevant for ICAM-1 and MCP-1, where we did not find any associations with severity. However, for these biomarkers, data did not show a pattern that could indicate an association with either ICC V or ICC N. Thus, we do not think that the lack of power calculations hampered the study.

To get a complete representation of the nerve injury, it is advised to perform a test battery of different sensory modalities that captures both small and large nerve fiber dysfunction^30^. Such dysfunctions are included in ICC N. A strength of the study is that we used both vibration perception thresholds and ICC. Vibration perception thresholds gave continuous data for severity of neurosensory manifestations, but an isolated small nerve fiber neuropathy was not captured by this test.

For vibration exposure, we did frequency weighting according to the standard ISO 5349-1, which underestimates the harm of higher frequencies or the use of impact tools^28,31^. This warrants further investigation in a prospective study.

Serum levels of TM were positively associated with the severity of RP, which is in line with a previous study on vibration-injured individuals, where a combination of SWS SN and SWS V was used^20^. Plasma levels of TM have also been shown to be elevated in hand-arm vibration injured patients compared to controls^20,32,33^. TM is an endothelial surface protein, normally expressed at low levels in serum, thus, this elevation might reflect a pathological condition in the endothelial cells due to vibration exposure. Elevated blood levels of TM have been associated with endothelial injury, e.g., in cardiovascular -, inflammatory-, infectious -, and metabolic diseases^34^.The association between serum levels of vWf and the severity of RP contrasts with a previous study, where vibration exposed workers with RP showed significantly lower blood concentration of vWf than vibration exposed controls without RP. This finding might reflect an ongoing mechanism with endothelial dysfunction, inflammation, and intimal hyperplasia. Hypothetically, increased levels of vWf could be caused by vibration-induced changes in shear stress and cyclic stretch to the vessel wall^35,36^. Increased vWf levels have been reported to shear stress in a dose–response relationship to intimal hyperplasia^37^. Furthermore vWf might reflect a state of inflammation, since individuals with RP, that later on developed a systemic connective tissue disease, showed elevated levels of vWf compared to individuals with only the primary form of RP^38^.

Interestingly, serum levels of CGRP were positively associated with both the vascular and the neurosensory components of hand-arm vibration injury. Hypothetically, injury can start in the small nerve fibers innervating blood vessels (nervi vasori) or in the small blood vessels supplying nerves (vasa nervorum)^39^, a pathophysiological mechanism described in diabetes neuropathy^40^. Structural changes in both blood vessels and nerves have been observed in relation to vibration exposure e.g., disrupted endothelial cell lining^9^ and loss of CGRP-containing nerve fibers^41^. A loss in CGRP-containing nerves have also been shown in patients with RP^42^. CGRP, a neuropeptide and very potent vasodilator, is released from unmyelinated small C-fibres and myelinated small Aδ-fibres^43^. CGRP exerts its effect mostly locally, but studies have shown that serum can be used in biomonitoring, e.g., elevated levels in serum have been shown in individuals in relation to episodes of migraine^44^.

Associations with HSP27 and caspase-3 in hand-arm vibration injury has to our knowledge not been presented earlier. In the present material, serum levels were also elevated compared to controls^3^. HSP27 act as chaperone to protect nerve structures under stress^45–47^ and increased levels of caspase-3 have been measured as an apoptotic response in Schwann cells, to balance the proliferative response after a nerve injury^48^.

Among the present patients, the prevalence of RP increased with rising severity of the neurosensory component and there was a great overlap between the three component; RP, small-, and large fibre neuropathy^49^. Thus, whether these biomarkers reflect an injury related to the vascular and/or the neurosensory component cannot be further elucidated in this study.

Several of the biomarker levels did not seem to be more elevated in ICC 3 V compared to ICC 2 V, indicating mechanisms where dysfunctional or injured endothelial cells first leads to unregulated release, measured as an increase of substances or compensatory mechanisms (stage 2)^43^. After repeated trauma, the endothelial cells or neuropeptidergic release from nerve fibres is diminished due to loss^41–43,50^ and might not be able to produce more substance, reflected by the levels at stage 3. Finally, a possible interplay between the observed biomarkers could further be a fact to take into account, e.g., treatment with CGRP has been shown to significantly decreased the expression of caspase-3^51^.

A previous study reported elevated plasma levels of ICAM-1 in individuals with rising severity of hand-arm vibration injury^20^, but this was not shown in the present study. For MCP-1 there was neither any association with the severity of hand arm vibration injury, nor the exposure.

## Conclusion

Levels of several serum biomarkers were associated with the severity gradings of hand-arm vibration injury. No associations with exposure were shown for these biomarkers. These findings give insight into pathophysiological mechanisms that can be useful in developing prospect tools for diagnostics, grading and treatment of hand-arm vibration injury.

## Methods

### Study design

The study has an observational case series design. Data presented in this study has been collected in previously described studies^3,49^. The scheme of the studies is presented in Fig. 1. All participants gave their informed consent to participate in the study before taking part. The study was conducted in accordance with the Declaration of Helsinki, and approved by the Regional Ethics Board in Lund, Sweden (No. 2018/15).

### Study group

Data were collected from patients consecutively referred to the Clinic for Occupational and Environmental Medicine in Lund, Sweden (enrolment from August 2018 until February 2020), following a standardized assessment for hand-arm vibration injury. When 100 patients had agreed to participate in the study, eight patients (seven men and one woman) declined to participate), one woman and seven men were considered by the physician not to have the diagnosis hand-arm vibration injury, and were therefore excluded, resulting in a study group of 92 patients, six of which were women^3,49^.

### Clinical assessment

A questionnaire was to be filled in before their visit and then the complete consultation and examination by a nurse, a physician and an occupational hygienist took approximately four hours^49^.

#### Questionnaire

The questionnaire included six questions on symptoms, where the responses (Yes) or (No) were indicated separately for the right and left hand: Do you experience: (a) numbness or tingling?, (b) numbness or tingling during the night?, (c) pain/discomfort in fingers/hands during cold exposure?, (d) white fingers when exposed to cold or dampness?, (e) poor grip strength?, (f) poor fine motor skills or clumsiness?.

Patients who reported that they experienced episodes of finger blanching were asked to fill in a hand diagram marking affected phalanges on both hands.

#### Clinical examination

Quantitative (monofilament and vibration perception threshold for large nerve fibers) and qualitative (temp rolls and pin prick test for small fibers) sensory tests were applied. Details on the outcome assessments are presented in the former study^49^. Before starting the tests, finger skin temperature was measured in second and fifth fingers bilaterally with a Testo 845 infrared measuring instrument. If the finger temperature was below 28 °C, the patient was asked to perform a shortened submaximal cycle ergometer test. Perception of vibration was tested with a VibroSense Meter I^®^. The patient was asked to wear earmuffs as high amplitudes at high frequencies generate an audible sound. Vibration perception thresholds were recorded at seven frequencies from 8 to 500 Hz^52^. The equipment automatically plots the data and generates a sensibility index (SI) defined as the ratio between the area under the test curve and that under an age-specific reference curve. Patients with a sensibility index below 0.8 were considered to have impaired vibrotactile sense^53^. Test of two-point discrimination (2PD) was used to assess tactile gnosis, and Purdue pegboard test was used to test manual dexterity.

#### Grading of severity of injury

The vascular manifestations were graded according to the ICC 0-3 V^22^ and were assessed using the Griffin score to grade finger blanching^23^. Blanching of the distal phalanx on the index, long, ring and little fingers corresponded to one point, middle phalanx: two, proximal phalanx: three points, and thumbs: four and five points for the distal and proximal phalanges. Scoring was done separately for the right and left hand. No episodic blanching of phalanges corresponded to stage 0 V, score 1–4: stage 1 V, score 5–12: stage 2 V, and score > 12: stage 3 V. Ten individuals reported episodes of finger blanching but had not filled in the hand-diagram. These were not included in the evaluation.

Severity of the neurosensory manifestations was graded separately for the hands, according to ICC 0-3N^22,49^. Stage N0: exposed to vibration but no numbness or tingling; stage 1N: numbness and/or tingling; stage 2N: as in 1N and impairment in two out of three sensory modalities; perception of touch, vibration, or temperature; stage 3N: as in 2N and symptoms of impaired dexterity and impaired manipulative dexterity.

### Biomarkers

Blood samples were collected after clinical examination, before noon, in 7 ml serum separation tubes with gel. After 30 min, serum was removed by centrifugation at 2000×*g* for 10 min, and the samples were stored at − 80 °C. Serum concentrations of TM, vWf, CGRP, HSP27 and caspase-3 were determined using commercially available ELISA kits, following the instructions provided by the manufacturers (TM from BioVendor, Brno, Czech Republic; vWf from Abnova, Taipei City; CGRP from FineTest, Hubei, China Taiwan; HSP27 from Millipore, Saint Louis, MO, USA; and caspase-3 from RayBiotech, Norcross, GA, USA)^3^. The intra- and inter-assay coefficient of variation was 3.9% and 9.8% (TM), 5.0% and 7.1% (vWf), < 8% and < 10% (CGRP), < 10% and < 12% (HSP27 and caspase-3).Serum concentrations of ICAM-1 and MCP-1 were determined by multiplexed immunoassays on a Luminex platform, both from Bio-Rad (Hercules, CA, USA). The intra- and inter-assay coefficient of variation was < 15% and < 25% (ICAM-1 and MCP-1).

### Vibration exposure

Exposure to vibration was assessed by a trained occupational hygienist^49^. Most of the patients were employed in the building industry, others in e.g., demolition, repair, dentistry, and gardening. The patients reported which tools they used in their current occupation and associated exposure time for each tool. The daily vibration exposure [A(8); (m/s2)] was calculated from the tools’ vibration magnitude and exposure time. Duration of vibration exposure in years was assessed.

### Statistics

SPSS IBM Statistics for Windows, Version 28.0 (IBM Corp., Armonk, NY, USA) was used for all statistical analysis. Serum levels of biomarkers were found not to be normally distributed, why they were presented as medians with interquartile ranges. Samples with results below the limit of detection (LOD) were awarded a value equal to half the LOD in the statistical analyses. ICC gradings, Griffin score and sensibility index (SI) were based on evaluation of the hand with most pronounced symptoms (rendering the highest score).

Jonckheere`s trend tests were performed and presented with p-values for possible associations between serum levels of biomarkers and severity of vascular and neurosensory manifestations according to ICC vascular and neural staging. Only two patients had ICC grade 0N, why these were excluded from the analyses.

Adjustment for possible confounders were assessed with generalized linear model (GLM) to estimate possible linear associations between serum level of each biomarker and severity of: (1) RP assessed as the extent of finger blanching (Griffin score), (2); neurosensory manifestations assessed as sensibility index (SI) for vibration perception thresholds; (3) current vibration exposure [A(8); (m/s^2^)] and; (4) duration of vibration exposure (years). The residuals were upon visual inspection of the p–p plots found not to be normally distributed. By using natural log transformed values we found the distribution acceptable for all biomarkers except for CGRP, thus not included in the GLM. The crude associations, β with 95% confidence intervals, were presented.

For each biomarker, associations between serum level and five potential confounders were tested: presence of concurrent diseases (yes/no), previous frostbites (yes/no), ongoing smoking (yes/no), age (years), and sex (male/female), one at a time by the GLM. Confounders with *p* < 0.10 were included in adjusted associations. Possible interactions (effect modifications) were tested by introducing an interaction term between the potential confounder and each of the four factors listed above (severity and exposure). When an interaction term with *p* value < 0.05 was found, the confounder was not included in the GLM model, instead data was dichotomized based on the effect modifier (for age < 45 year and ≥ 45 years).

### Ethics approval

The study was conducted in accordance with the Declaration of Helsinki, and approved by the Regional Ethics Board in Lund, Sweden (No. 2018/15).

### Informed consent

Participants gave their informed consent to participate in the study before taking part.

## Data Availability

The datasets presented in this article are not readily available as public access to data is restricted to Swedish Authorities (Public Access to Information and Secrecy Act), but data can be made available for researchers after a special review that includes approval of the research project by both an Ethics Committee and the Authorities’ Data Safety Committees. Contact the corresponding author for request.

## References

[1] Nilsson, T., Wahlström, J. & Burström, L. Hand-arm vibration and the risk of vascular and neurological diseases—a systematic review and meta-analysis. *PLoS ONE* **12**, e0180795–e0180795 (2017). 28704466 10.1371/journal.pone.0180795 PMC5509149

[2] Heaver, C., Goonetilleke, K. S., Ferguson, H. & Shiralkar, S. Hand-arm vibration syndrome: A common occupational hazard in industrialized countries. *J. Hand Surg. Eur.* **36**, 354–363 (2011). 10.1177/1753193410396636 21310765

[3] Tekavec, E. *et al.* Serum biomarkers in patients with hand-arm vibration injury and in controls. *Sci. Rep.* **14**, 2719 (2024). 38302542 10.1038/s41598-024-52782-1 PMC10834969

[4] Dahlin, L. B. *et al.* Low myelinated nerve-fibre density may lead to symptoms associated with nerve entrapment in vibration-induced neuropathy. *J. Occup. Med. Toxicol.* **9**, 7 (2014). 24606755 10.1186/1745-6673-9-7 PMC3974023

[5] Takeuchi, T., Takeya, M. & Imanishi, H. Ultrastructural changes in peripheral nerves of the fingers of three vibration-exposed persons with Raynaud’s phenomenon. *Scand. J. Work Environ. Health* **14**, 31–35 (1988). 3353694 10.5271/sjweh.1953

[6] Takeuchi, T., Futatsuka, M., Imanishi, H. & Yamada, S. Pathological changes observed in the finger biopsy of patients with vibration-induced white finger. *Scand. J. Work Environ. Health* **12**, 280–283 (1986). 3775312 10.5271/sjweh.2140

[7] Strömberg, T., Dahlin, L. B., Brun, A. & Lundborg, G. Structural nerve changes at wrist level in workers exposed to vibration. *Occup. Environ. Med.* **54**, 307–311 (1997). 9196451 10.1136/oem.54.5.307 PMC1128777

[8] Govindaraju, S. R., Curry, B. D., Bain, J. L. & Riley, D. A. Comparison of continuous and intermittent vibration effects on rat-tail artery and nerve. *Muscle Nerve* **34**, 197–204 (2006). 16691604 10.1002/mus.20578

[9] Wei, N. *et al.* Local vibration induced vascular pathological structural changes and abnormal levels of vascular damage indicators. *Microvasc. Res* **136**, 104163 (2021). 33831407 10.1016/j.mvr.2021.104163

[10] Ekenvall, L. & Lindblad, L. E. Is vibration white finger a primary sympathetic nerve injury?. *Br. J. Ind. Med.* **43**, 702–706 (1986). 2877686 10.1136/oem.43.10.702 PMC1007740

[11] Harada, N., Kondo, H. & Kimura, K. Assessment of autonomic nervous function in patients with vibration syndrome using heart rate variation and plasma cyclic nucleotides. *Br. J. Ind. Med.* **47**, 263–268 (1990). 2159773 10.1136/oem.47.4.263 PMC1035148

[12] Laskar, M. S. *et al.* Heart rate variability in response to psychological test in hand-arm vibration syndrome patients assessed by frequency domain analysis. *Ind. Health* **37**, 382–389 (1999). 10547953 10.2486/indhealth.37.382

[13] Stoyneva, Z., Tzvetkov, D., Vodenicharov, E. & Lyapina, M. Current pathophysiological views on vibration-induced Raynaud’s phenomenon. *Cardiovasc. Res.* **57**, 615–624 (2003). 12618223 10.1016/S0008-6363(02)00728-9

[14] Krajnak, K. & Waugh, S. Systemic effects of segmental vibration in an animal model of hand-arm vibration syndrome. *J. Occup. Environ. Med.* **60**, 886–895 (2018). 30020212 10.1097/JOM.0000000000001396 PMC6173648

[15] Laskar, M. S. & Harada, N. Assessment of autonomic nervous activity in hand-arm vibration syndrome patients using time- and frequency-domain analyses of heart rate variation. *Int. Arch. Occup. Environ. Health* **72**, 462–468 (1999). 10541911 10.1007/s004200050399

[16] Laskar, S. M., Iwamoto, M., Nakamoto, M., Koshiyama, H. & Harada, N. Heart rate variation and urinary catecholamine excretion in response to acute psychological stress in hand-arm vibration syndrome patients. *J. Occup. Health* **46**, 125–131 (2004). 15090687 10.1539/joh.46.125

[17] Blann, A. D., Herrick, A. & Jayson, M. I. V. Altered levels of soluble adhesion molecules in rheumatoid arthritis, vasculitis and systemic sclerosis. *Rheumatology* **34**, 814–819 (1995). 10.1093/rheumatology/34.9.814 7582719

[18] Gruschwitz, M. S., Hornstein, O. P. & Driesch, P. V. D. Correlation of soluble adhesion molecules in the peripheral blood of scleroderma patients with their in situ expression and with disease activity. *Arthr. Rheu.* **38**, 184–189 (1995). 10.1002/art.1780380206 7848308

[19] Kennedy, Khan, McLaren, & Belch,. Endothelial activation and response in patients with hand arm vibration syndrome. *Eur. J. Clin. Investig.* **29**, 577–581 (1999). 10411662 10.1046/j.1365-2362.1999.00502.x

[20] Kao, D. S. *et al.* Serological tests for diagnosis and staging of hand-arm vibration syndrome (HAVS). *Hand (N. Y.)* **3**, 129–134 (2008). 18780088 10.1007/s11552-007-9079-6 PMC2529134

[21] Brammer, A. J., Taylor, W. & Lundborg, G. Sensorineural stages of the hand-arm vibration syndrome. *Scand. J. Work Environ. Health* **13**, 279–283 (1987). 3324308 10.5271/sjweh.2050

[22] Poole, C. J. M. *et al.* International consensus criteria for diagnosing and staging hand-arm vibration syndrome. *Int. Arch. Occup. Environ. Health* **92**, 117–127 (2019). 30264331 10.1007/s00420-018-1359-7 PMC6323073

[23] Griffin, M. J. & Erdreich, J. *Handbook of Human Vibration* (Acoustical Society of America, 1991).

[24] Welcome, D. E., Krajnak, K., Kashon, M. L. & Dong, R. G. An investigation on the biodynamic foundation of a rat tail vibration model. *Proc. Inst. Mech. Eng. Part H J. Eng. Med.* **222**, 1127–1141 (2008). 10.1243/09544119JEIM419 19024160

[25] Pacurari, M., Waugh, S. & Krajnak, K. Acute vibration induces peripheral nerve sensitization in a rat tail model: Possible role of oxidative stress and inflammation. *Neuroscience* **398**, 263–272 (2019). 30553794 10.1016/j.neuroscience.2018.12.010 PMC6545897

[26] Krajnak, K. M. *et al.* The effects of impact vibration on peripheral blood vessels and nerves. *Ind. Health* **51**, 572–580 (2013). 24077447 10.2486/indhealth.2012-0193 PMC4202742

[27] Goenka, S., Peelukhana, S. V., Kim, J., Stringer, K. F. & Banerjee, R. K. Dependence of vascular damage on higher frequency components in the rat-tail model. *Ind. Health* **51**, 373–385 (2013). 23518603 10.2486/indhealth.2012-0060

[28] Raju, S. G., Rogness, O., Persson, M., Bain, J. & Riley, D. Vibration from a riveting hammer causes severe nerve damage in the rat tail model. *Muscle Nerve* **44**, 795–804 (2011). 22006694 10.1002/mus.22206

[29] Curry, B. D. *et al.* Evidence for frequency-dependent arterial damage in vibrated rat tails. *Anat. Rec. Part A Discov. Mol. Cell. Evol. Biol.* **284A**, 511–521 (2005). 10.1002/ar.a.20186 15791580

[30] Rolke, R. *et al.* Hand-arm vibration syndrome: Clinical characteristics, conventional electrophysiology and quantitative sensory testing. *Clin. Neurophysiol.* **124**, 1680–1688 (2013). 23507585 10.1016/j.clinph.2013.01.025

[31] Bovenzi, M. & Tarabini, M. Cold response of digital vessels and metrics of daily vibration exposure. In *Proceedings*, Vol. 86 (2023).

[32] Toibana, N., Kanazuka, M. & Shigekiyo, T. High level of plasma thrombomodulin (TM) concentration and correlation with endothelin (ET)-1 in vibration-exposed patients. *Central Eur. J. Public Health* **3**(Suppl), 40–42 (1995). 9150966

[33] Kanazuka, M., Shigekiyo, T., Toibana, N. & Saito, S. Increase in plasma thrombomodulin level in patients with vibration syndrome. *Thromb. Res.* **82**, 51–56 (1996). 8731509 10.1016/0049-3848(96)00050-3

[34] Boron, M., Hauzer-Martin, T., Keil, J. & Sun, X. L. Circulating thrombomodulin: Release mechanisms, measurements, and levels in diseases and medical procedures. *TH Open* **6**, e194–e212 (2022). 36046203 10.1055/a-1801-2055 PMC9273331

[35] Noe̋l, C. & Settembre, N. Assessing mechanical vibration-altered wall shear stress in digital arteries. *J. Biomech.* **131**, 110893 (2022). 34953283 10.1016/j.jbiomech.2021.110893

[36] van Haaften, E. E., Wissing, T. B., Kurniawan, N. A., Smits, A. I. P. M. & Bouten, C. V. C. Human in vitro model mimicking material-driven vascular regeneration reveals how cyclic stretch and shear stress differentially modulate inflammation and matrix deposition. *Adv. Biosyst.* **4**, 1900249 (2020). 10.1002/adbi.201900249 32390338

[37] Qin, F., Impeduglia, T., Schaffer, P. & Dardik, H. Overexpression of von Willebrand factor is an independent risk factor for pathogenesis of intimal hyperplasia: Preliminary studies. *J. Vasc. Surg.* **37**, 433–439 (2003). 12563218 10.1067/mva.2003.63

[38] Gualtierotti, R. *et al.* Detection of early endothelial damage in patients with Raynaud’s phenomenon. *Microvasc. Res.* **113**, 22–28 (2017). 28450106 10.1016/j.mvr.2017.04.004

[39] Glatte, P., Buchmann, S. J., Hijazi, M. M., Illigens, B. M. & Siepmann, T. Architecture of the cutaneous autonomic nervous system. *Front. Neurol.* **10**, 970 (2019). 31551921 10.3389/fneur.2019.00970 PMC6746903

[40] Cameron, N. E., Eaton, S. E. M., Cotter, M. A. & Tesfaye, S. Vascular factors and metabolic interactions in the pathogenesis of diabetic neuropathy. *Diabetologia* **44**, 1973–1988 (2001). 11719828 10.1007/s001250100001

[41] Goldsmith, P. C. *et al.* Cutaneous nerve fibre depletion in vibration white finger. *J. R. Soc. Med.* **87**, 377–381 (1994). 8046721 10.1177/014107689408700703 PMC1294645

[42] Bunker, C. B., Terenghi, G., Springall, D. R., Polak, J. M. & Dowd, P. M. Deficiency of calcitonin gene-related peptide in Raynaud’s phenomenon. *Lancet* **336**, 1530–1533 (1990). 1979366 10.1016/0140-6736(90)93307-B

[43] Russell, F. A., King, R., Smillie, S. J., Kodji, X. & Brain, S. D. Calcitonin gene-related peptide: Physiology and pathophysiology. *Physiol. Rev.* **94**, 1099–1142 (2014). 25287861 10.1152/physrev.00034.2013 PMC4187032

[44] Gárate, G. *et al.* Serum alpha and beta-CGRP levels in chronic migraine patients before and after monoclonal antibodies against CGRP or its receptor. *Ann. Neurol.* **94**, 285–294 (2023). 37038806 10.1002/ana.26658

[45] de Los Reyes, T. & Casas-Tintó, S. Neural functions of small heat shock proteins. *Neural Regen. Res.* **17**, 512–515 (2022). 34380880 10.4103/1673-5374.320975 PMC8504394

[46] Ising, E. *et al.* Quantitative proteomic analysis of human peripheral nerves from subjects with type 2 diabetes. *Diabetic Med.* **38**, e14658 (2021). 34309080 10.1111/dme.14658

[47] Vidyasagar, A., Wilson, N. A. & Djamali, A. Heat shock protein 27 (HSP27): Biomarker of disease and therapeutic target. *Fibrogenes. Tissue Repair* **5**, 7 (2012). 10.1186/1755-1536-5-7 PMC3464729 22564335

[48] Dahlin, L. B. The dynamics of nerve degeneration and regeneration in a healthy milieu and in diabetes. *Int. J. Mol. Sci.* **24**, 15241 (2023). 37894921 10.3390/ijms242015241 PMC10607341

[49] Tekavec, E., Nilsson, T., Riddar, J., Axmon, A. & Nordander, C. Concordance between the Stockholm Workshop Scale and the International Consensus Criteria for grading the severity of neurosensory manifestations in hand-arm vibration syndrome in a Swedish clinical setting. *Occup. Environ. Med.* **80**, 418–424 (2023). 37193594 10.1136/oemed-2023-108914

[50] Bunker, C. B. *et al.* Calcitonin gene-related peptide, endothelin-1, the cutaneous microvasculature and Raynaud’s phenomenon. *Br. J. Dermatol.* **134**, 399–406 (1996). 8731660 10.1111/j.1365-2133.1996.tb16221.x

[51] Luo, C.-C., Huang, C.-S., Ming, Y.-C., Chu, S.-M. & Chao, H.-C. Calcitonin gene-related peptide downregulates expression of inducible nitride oxide synthase and caspase-3 after intestinal ischemia-reperfusion injury in rats. *Pediatr. Neonatol.* **57**, 474–479 (2016). 27117955 10.1016/j.pedneo.2015.10.012

[52] Strömberg, T., Dahlin, L. B. & Lundborg, G. Vibrotactile sense in the hand-arm vibration syndrome. *Scand. J. Work Environ. Health* **24**, 495–502 (1998). 9988092 10.5271/sjweh.374

[53] Lundborg, G., Dahlin, L. B., Lundstrom, R., Necking, L. E. & Stromberg, T. Vibrotactile function of the hand in compression and vibration-induced neuropathy. Sensibility index—a new measure. *Scand. J. Plast. Reconstr. Surg. Hand* **26**, 275–279 (1992). 10.3109/02844319209015271 1335164

